# The Impact of Emergency Department Process Optimization Combined With Health Education on the Treatment Outcomes and Anxiety and Depression in Patients With Acute Ischemic Stroke

**DOI:** 10.62641/aep.v54i3.2175

**Published:** 2026-06-15

**Authors:** Xiaodong Xie, Shouzhang Wang, Mingying Zhang, Lifen Tang

**Affiliations:** ^1^Emergency Department, Zhejiang Sian International Hospital, 314000 Jiaxing, Zhejiang, China; ^2^Neurology Department, Zhejiang Sian International Hospital, 314000 Jiaxing, Zhejiang, China

**Keywords:** acute ischaemic stroke, emergency department process optimisation, health education, anxiety and depression

## Abstract

**Background:**

To explore the association between an optimised emergency procedure combined with structured health education and anxiety, depression and treatment outcomes in patients with acute ischaemic stroke (AIS).

**Methods:**

We conducted a retrospective cohort study of 114 AIS patients admitted to our stroke centre between January and December 2023. On the basis of the admission timeframe, patients were divided into a conventional care group (n = 57, January to June 2023, routine care) and a comprehensive nursing group (n = 57, July to December 2023, optimised integrated care). Emergency nursing outcomes were assessed after stabilisation and before ward transfer. Neurological recovery was assessed with the European Stroke Scale (ESS); anxiety and depression were assessed with the Generalized Anxiety Disorder-7 (GAD-7) and Patient Health Questionnaire-9 (PHQ-9) as primary psychological indicators. Process times and nursing satisfaction were also evaluated. Data analysis was conducted using SPSS 27.0. Normality was verified using the Shapiro-Wilk test. Normally distributed data were expressed as mean ± standard deviation and analysed using an independent samples *t*-test, whereas non-normal data were expressed as median (interquartile range) and analysed using the Mann-Whitney U test. Categorical data were analysed using chi-square test. Multivariate linear and logistic regression were used to analyse indicators such as neurological function and nursing satisfaction, adjusting for confounding factors. A *p*-value < 0.05 was considered statistically significant.

**Results:**

Compared with the conventional care group, the waiting time, diagnosis time, emergency room waiting time and recanalisation time of patients in the comprehensive nursing group were significantly shortened (*p* < 0.05). The degree of improvement in ESS (ΔESS) in the comprehensive nursing group was significantly higher, and the reductions in GAD-7 and PHQ-9 scores were also significantly greater (*p* < 0.05). Patient and family satisfaction in the comprehensive nursing group were significantly higher than those in the conventional care group (*p* < 0.05). Through multivariate linear regression analysis, with baseline National Institutes of Health Stroke Scale (NIHSS) score, time from onset to visit, hypertension and diabetes as adjustment factors, allocation to the comprehensive nursing group was still significantly correlated with ΔESS (β [95% confidence interval (CI)] = 8.15 [5.72 to 10.58], *p* < 0.001), ΔGAD-7 (β [95% CI] = −2.18 [−2.92 to −1.44], *p* < 0.001), and ΔPHQ-9 (β [95% CI] = −2.65 [−3.32 to −1.98], *p* < 0.001). The results of multivariate logistic regression analysis showed that, after adjusting for the above confounding factors, inclusion in the comprehensive nursing group was independently associated with a significantly higher likelihood of obtaining a satisfactory nursing evaluation (adjusted OR = 8.915, 95% CI: 2.453 to 32.468, *p* = 0.001).

**Conclusions:**

The integrated model is associated with shorter treatment times, better neurological recovery, reduced anxiety and depression and higher satisfaction in AIS patients, providing preliminary evidence for a patient-centred comprehensive emergency care model.

## Introduction

Acute ischaemic stroke (AIS) is a central nervous system vascular event caused by cerebral artery occlusion leading to cerebral infarction, accompanied by damage to neurons, astrocytes and oligodendrocytes [1]. As a major cause of disability and death, it still places a significant burden on global public health and is an important cause of death and disability in modern society [2, 3]. The treatment effectiveness of AIS is highly dependent on time. The time window from onset to vascular recanalisation is very short. Delay in treatment will directly lead to irreversible death of nerve cells in the ischaemic penumbra, seriously affecting the recovery of neurological function and long-term quality of life of patients [4]. Currently, although stroke green channels have been widely established in medical institutions at all levels, problems such as poor pre-hospital and in-hospital coordination, low efficiency of multidisciplinary collaboration within hospitals and excessively long time consumption in the examination and treatment process still exist in actual operation. These issues restrict the further improvement of treatment efficiency. In addition to physical injury, AIS can have an adverse impact on patients’ mental health, with the most prominent psychological sequelae being the simultaneous occurrence of depression and anxiety [5, 6] . These negative psychological states not only reduce patients’ treatment compliance, affect doctor-patient communication and increase the medical burden, but also have a negative impact on patients’ functional recovery, becoming an important negative factor affecting the overall treatment effect [7, 8]. Traditional emergency nursing models often focus on technical operations and disease management, with insufficient attention to systematic health education and psychological support for patients and their families, making it difficult to meet their overall physical and mental needs [9, 10]. Currently, research on improving AIS care quality primarily progresses along two separate tracks. One track focuses on technical process re-engineering, such as optimising green channels and promoting multidisciplinary collaboration to reduce treatment delays. The other track addresses the integrated care pathway for patient psychology, such as using health education to alleviate post-stroke anxiety and depression [11]. However, studies that systematically integrate process optimisation with structured health education and comprehensively evaluate their dual impact on treatment efficiency and patient psychological well-being remain scarce. This integrated care pathway model may create a synergistic effect: it can improve biological prognosis by enhancing efficiency while also promoting psychological recovery by increasing patient understanding and sense of control. This approach aligns with the modern concept of “patient-centred” comprehensive stroke management. Even so, few studies have systematically integrated these two aspects into a cohesive care pathway and evaluated their combined impact on hard clinical endpoints and patient-reported psychological outcomes. To address this gap, this study, guided by patient-centred care and social cognitive theory, aims to investigate and evaluate an integrated model combining process optimisation with structured psychoeducation, and to explore its impact on treatment time, neurological function recovery and anxiety and depression in patients with AIS.

## Materials and Methods

### General Information

A retrospective cohort study was conducted, and 156 patients with AIS who were scheduled to be included in the study at our stroke centre from January to December 2023 were selected. The inclusion criteria for this study were: (1) meeting the AIS diagnosis criteria in the “Chinese Guidelines for the Diagnosis and Treatment of Acute Ischaemic Stroke 2018” [12], and confirmed by head CT or Magnetic Resonance Imaging (MRI); (2) the time from onset to admission was < 24 hours; (3) age ≥ 18 years; (4) complete clinical data were available for research analysis. Exclusion criteria were as follows: (1) diagnosis of cerebral haemorrhage or other non-ischaemic neurological diseases after admission; (2) coma at admission (Glasgow Coma Scale score ≤8); (3) severe dementia, a history of mental illness or advanced malignant tumours; (4) missing > 0% of key time points or assessment data. Their clinical data were evaluated for eligibility. Among them, 21 patients were excluded because they were diagnosed with cerebral haemorrhage or other non-ischaemic neurological diseases after admission, 9 patients were excluded because they were in a coma (Glasgow Coma Scale score ≤8), 7 patients were excluded because they had severe dementia, a history of mental illness or advanced malignant tumours, and 5 patients were excluded because the key time points or assessment data were missing (>20%). Finally, 114 patients were included in the analysis of this study. They were divided into the comprehensive nursing group (57 cases) and the conventional care group (57 cases) on the basis of the admission time. The conventional care group consisted of AIS patients who were admitted consecutively from January to June 2023, during which the hospital implemented the standard emergency treatment plan. The comprehensive nursing group consisted of AIS patients who were admitted consecutively from July to December 2023, during which the hospital had officially implemented the integrated and optimised care pathway.

All patients or their families signed an informed consent form. This study adopted a blinded assessment design. The research subjects and the outcome assessors were unaware of the group allocation and intervention information. They voluntarily participated in the study and agreed to the use and analysis of the relevant clinical data. The informed consent form clearly explained the principles of the blinded design, the usage norms of the research data and the potential benefits and risks of the study. This study obtained approval from the Hospital Ethics Committee (XA-K-2025-007) and was conducted in accordance with the Declaration of Helsinki. The grouping information of the study was concealed using sealed envelopes. Only the data management specialist had access to the grouping list. The outcome assessors and clinical care providers were strictly separated throughout the process, maintaining full blinding.

### Integrated Care Pathway Methods

#### Conventional Care Group (Conventional Sequential Care Pathway

Patients who received care according to the hospital’s standard, linear emergency protocol for suspected stroke were categorised into the control group. This pathway was characterised by sequential, stepwise execution of tasks: beginning with standard triage and registration, followed by an initial assessment by an emergency department (ED) physician and a separate consultation request to the on-duty neurologist. Upon the neurologist’s orders, laboratory tests and a non-contrast head CT scan were then performed sequentially, with the patient travelling between departments. After returning to the ED to await results, the CT images and lab reports were reviewed by physicians in turn. Once a diagnosis was confirmed and eligibility for reperfusion therapy was determined, the treating physician conducted a separate discussion with the patient and family to obtain informed consent. Only after consent was secured did the preparation and administration of intravenous thrombolysis or transfer for thrombectomy commence. Throughout this process, health communication was informal, variable and reactive; information was provided sporadically by different staff members in response to questions, without standardised content, format or a dedicated provider.

#### Comprehensive Nursing Group (Integrated and Optimised Care Pathway)

Patients who received the protocolised, integrated care pathway designed to minimise delays and address psychological needs were categorised into the comprehensive nursing group. This pathway featured parallel processing and proactive, structured support. Upon a pre-hospital stroke alert or identification at triage, a dedicated team—comprising an ED physician, neurologist, stroke nurse and radiology technician—was activated simultaneously via a one-call system. The patient was taken directly to the CT scanner via a pre-cleared route, and critical tasks were performed in parallel. Intravenous access was established and blood samples were drawn while the patient was on the CT table, the CT scan was performed immediately and the neurologist and radiologist reviewed images in real time via a shared workstation. Using a standardised decision-support algorithm, the team made a rapid treatment recommendation, and a structured, concise conversation led by the neurologist and stroke nurse combined diagnosis explanation, treatment options and consent acquisition into a single, efficient interaction. If consent was obtained, thrombolytic medication was administered in the CT suite or the patient was transferred directly to the angiography lab. Integral to this pathway was structured health education and psychological first aid delivered by the assigned Stroke Nurse Coordinator across three phases. During the acute phase, brief, clear explanations were provided using simple language and visual aids about “time is brain” and immediate procedures. Within 24 hours of admission, comprehensive education was delivered using a booklet on stroke causes, medications and early rehabilitation. In the pre-discharge phase, the focus was on secondary prevention, warning signs, medication adherence and recovery coping strategies. This communication was proactive, standardised and empathetic, aimed at reducing uncertainty and fostering a sense of control.

### Observation Indicators

All outcome assessments were conducted at two predefined time points by trained research staff to ensure consistency. Baseline (T0) assessment was conducted within 24 hours of admission after initial stabilisation and prior to ward transfer, and outcome (T1) assessment was conducted within 24 hours before scheduled discharge. The European Stroke Scale (ESS), Self-Rating Anxiety Scale (SAS), Self-Rating Depression Scale (SDS), Generalized Anxiety Disorder-7 (GAD-7) and Patient Health Questionnaire-9 (PHQ-9) were administered at T0 and T1 to evaluate neurological function and psychological status. Process efficiency time metrics were extracted retrospectively from hospital system timestamps, and patient satisfaction was assessed at T1 only using a dedicated questionnaire.

All baseline data were systematically extracted by two trained researchers from the following sources within the hospital’s electronic medical record system: (1) Patient Demographics Module (for age and sex); (2) ED and Admission Notes (for time of symptom onset, time of arrival and initial neurological assessment); (3) Past Medical History and Problem List (for comorbidities such as hypertension, diabetes, atrial fibrillation, coronary artery disease and prior stroke/TIA); (4) Nursing Assessment Flowsheets (for height and weight, used to calculate body mass index (BMI)); (5) Medication Administration Records and Procedure Notes (for details of reperfusion therapy, i.e., intravenous thrombolysis and/or mechanical thrombectomy); (6) Neurological Specialty Assessment Records, extracting the National Institutes of Health Stroke Scale (NIHSS) score. This score was determined by a neurologist who has received professional training and is qualified. Within 24 hours after the patient’s admission (baseline T0), the neurologist followed the standardised assessment process of the NIHSS scale to conduct a detailed assessment and score each dimension of the patient’s consciousness, fixation, visual field, facial paralysis, limb movement, ataxia, sensation, language, articulation disorder and neglect. The assessment process strictly adhered to the operational norms of the scale to ensure the objectivity and consistency of the score.

Rationale for Scale Selection and Primary Psychological Indicators: The SAS and SDS were employed as established, broad-spectrum screening tools for anxiety and depression tendencies, commonly used in the Chinese clinical context. To provide more specific, DSM-aligned severity measurement with established clinical cut-offs, the GAD-7 and PHQ-9 scales were pre-specified as the primary indicators for quantifying the severity of anxiety and depressive symptoms, respectively, in this study.

#### ESS Score

The scale focuses on the quantitative assessment of motor function and level of consciousness. It is a 14-item scale evaluating consciousness, comprehension, speech, visual fields, gaze, facial palsy, arm (proximal and distal) motor power, leg motor power and gait. It employs a weighted scoring system where each item has a predefined maximum score (ranging from 2 to 8 points), and a higher score indicates better function. The scoring is structured such that a patient with normal neurological function in all assessed domains achieves the predefined maximum total score of 100. Thus, the total score ranges from 0 (worst possible deficit) to 100 (normal function). This scale was administered at admission (baseline) and at discharge (outcome) [13]. The ESS was selected as the primary neurological outcome at discharge because it provides a comprehensive and weighted assessment of functional domains relevant to post-stroke recovery, with higher sensitivity to change in the subacute phase compared to the NIHSS, which is more suited for assessing acute stroke severity. The minimal clinically important difference (MCID) for ESS in AIS is estimated to be around 5 to 10 points, making the observed group differences clinically meaningful.

#### SAS Score

The scale contains 20 items, covering core symptom dimensions such as anxious mood, autonomic dysfunction, motor tension and panic. It uses a 4-point scale (1 to 4 points) with a total score range of 20 to 80 points. The standard score is obtained by multiplying the total score by 1.25 and rounding it down. Clinically, 50 points is often used as the cut-off value for anxiety symptoms. The higher the score, the more severe the anxiety symptoms. The scale has good reliability and validity and is suitable for rapid screening and severity assessment of clinical anxiety symptoms [14]. In this study, its Cronbach’s α was approximately 0.85.

#### SDS Rating

The scale contains 20 items, covering core dimensions such as depressed mood, somatic symptoms, psychomotor disorders and psychological distress. It uses a 4-point scale (1 to 4 points) with a total score range of 20 to 80 points. The final standard score is obtained by multiplying the total score by 1.25 and rounding it down. Clinically, 53 points is often used as the cut-off value for depressive symptoms. The higher the score, the more significant the depressive tendency. The scale has good reliability and validity and is suitable for rapid clinical screening and preliminary assessment of symptom severity [14]. In this study, its Cronbach’s α was approximately 0.86.

#### GAD-7 Rating

Participants scored themselves based on the frequency of seven anxiety symptoms over the past two weeks, using a four-point scale of 0 to 3 (0 means “never” and 3 means “almost every day”). The total score ranged from 0 to 21, with 10 as the clinical cut-off value for distinguishing anxiety symptoms [15]. This cut-off value has been widely used in clinical studies at home and abroad and has good screening validity and clinical applicability. It can effectively identify people with clinically significant anxiety symptoms and provide a quantitative basis for early psychological support. In this study, the GAD-7 demonstrated good internal consistency (Cronbach’s α = 0.92).

#### PHQ-9 Rating

The scale contains nine items, each item is scored on a four-level scale of 0 to 3, and the total score ranges from 0 to 27. Participants are required to evaluate the degree of distress of the corresponding symptoms in the past two weeks. A total score of ≥10 is usually used as the clinical diagnostic threshold for depressive symptoms. It is one of the most widely used self-rating scales for depressive symptoms and is recommended by many clinical guidelines for quantifying the severity of depression [16]. In this study, the PHQ-9 demonstrated good internal consistency (Cronbach’s α = 0.89).

#### Assessment of Nursing Satisfaction

Patient and family satisfaction with the emergency and acute nursing care experience was evaluated using a self-developed, 10-item Emergency Nursing Satisfaction Questionnaire. The questionnaire items were constructed based on the core dimensions of patient-centred care. They covered: 1) clarity and timeliness of communication regarding the condition and procedures, 2) perceived professional competence and empathy of the nursing staff, 3) respect for patient privacy and dignity, 4) responsiveness to patient needs and concerns and 5) the overall environment and efficiency of the emergency care process. Each item was rated on a 5-point Likert scale: 1 = Very Dissatisfied, 2 = Dissatisfied, 3 = Neutral, 4 = Satisfied, 5 = Very Satisfied. The total satisfaction score is the sum of all item scores (range: 10 to 50). For categorical analysis, an overall response of “Satisfied” or “Very Satisfied” (item score ≥4) on ≥80% of the items was defined as a “Satisfied” evaluation. The questionnaire demonstrated good internal consistency in this sample (Cronbach’s α = 0.89).

#### Emergency Treatment Process Time Metrics

Key time intervals (measured in minutes) were calculated using timestamp data automatically recorded in the Hospital Information System (HIS) and the dedicated Stroke Green Channel Registry. A researcher, blinded to group allocation, extracted these timestamps for analysis. The metrics were defined as follows:

Waiting Time: Time from patient arrival at the ED registration desk to the time of initial assessment and triage completion by a nurse.

Diagnosis Time: Time from ED arrival to the time when a definitive diagnosis of AIS was recorded in the electronic medical record, following neuroimaging (CT/MRI) review and neurologist confirmation.

Emergency Room Waiting Time: Time from completion of triage to the time when the patient physically left the ED area for a dedicated inpatient stroke unit or the angiography suite.

Vascular Recanalisation Time: For patients receiving reperfusion therapy, this was defined as: a) Door-to-Needle Time: Time from ED arrival to the start of intravenous thrombolytic agent infusion or b) Door-to-Puncture Time: Time from ED arrival to the time of femoral artery puncture for mechanical thrombectomy.

### Statistical Methods

Data were analysed using SPSS Statistics software (Version 27.0; IBM Corp., Armonk, NY, USA). All statistical tests in this study were conducted using a two-tailed approach. The criterion for determining statistical significance was *p* < 0.05. Continuous variables were first tested for normality using the Shapiro-Wilk test. Normally distributed continuous variables were expressed as mean ± standard deviation and compared between groups using the independent samples *t*-test. Non-normally distributed continuous variables were expressed as median (interquartile range, IQR) and compared using the Mann-Whitney U test. Categorical variables were expressed as frequencies (n, %) and compared using the chi-square test, as appropriate. For outcome measures (ESS, SAS, SDS, GAD-7 and PHQ-9) assessed at admission and discharge, between-group comparisons were performed on the change scores (Δ = discharge score minus admission score) using independent samples *t*-tests or the Mann-Whitney U test, as appropriate. To adjust for potential residual confounding, multivariable linear regression models were constructed for the primary (ΔESS) and key secondary continuous outcomes (ΔGAD-7 and ΔPHQ-9), with the care pathway group as the main independent variable. Confounding variables (baseline NIHSS score, onset-to-door time, hypertension and diabetes) were selected based on clinical relevance and prior literature indicating their potential influence on stroke outcomes. A multivariable logistic regression model was used for the binary satisfaction outcome with the same adjustments. Given the exploratory nature of the other psychological scale comparisons (ΔSAS and ΔSDS), the results were reported without adjustment for multiple comparisons and should be interpreted with caution.

## Results

### General Characteristics

As shown in Table 1, the baseline demographic and clinical characteristics were well balanced between the conventional care group and the comprehensive nursing group. No statistically significant differences were observed in age, sex, BMI, time from symptom onset to arrival, admission stroke severity (NIHSS score), prevalence of key comorbidities or the distribution of reperfusion therapy modalities (all *p* > 0.05).

**Table 1.** **Baseline characteristics of the study and control groups**.

Characteristic		Conventional care group (n = 57)	Comprehensive nursing group (n = 57)	χ^2^/*t/Z*	*p*
Age (years, mean ± SD)		68.41 ± 10.13	66.93 ± 9.82	0.792	0.43
Male, n (%)		32 (56.14)	30 (52.63)	0.142	0.707
BMI (kg/m^2^, mean ± SD)		24.81 ± 3.52	25.06 ± 3.18	-0.398	0.691
Onset-to-door time (min)		130 (85, 210)	125 (80, 195)	0.432	0.666
Admission NIHSS score		7 (4, 12)	6 (4, 10)	1.024	0.306
Medical history, n (%)					
	Hypertension	40 (70.18)	43 (75.44)	0.399	0.528
	Diabetes mellitus	18 (31.58)	21 (36.84)	0.342	0.559
	Atrial fibrillation	12 (21.05)	9 (15.79)	0.526	0.468
	Coronary artery disease	10 (17.54)	8 (14.04)	0.264	0.607
	Prior stroke/TIA	8 (14.04)	6 (10.53)	0.326	0.568
Reperfusion therapy, n (%)				0.347	0.951
	Intravenous thrombolysis only	15 (26.32)	17 (29.82)		
	Mechanical thrombectomy only	10 (17.54)	8 (14.04)		
	Bridging therapy (IVT + MT)	5 (8.77)	5 (8.77)		
	Standard medical therapy	27 (47.37)	27 (47.37)		

Notes: BMI, body mass index; onset-to-door time, time from onset to admission; NIHSS, National Institutes of Health Stroke Scale; TIA, transient ischaemic attack; IVT, intravenous thrombolysis; MT, mechanical thrombectomy.

### Comparison of Emergency Treatment Efficiency

The comprehensive nursing group was associated with significantly shorter waiting times, diagnosis times, emergency room waiting times and recanalisation times than the control group, with statistically significant differences (*p* < 0.001). See Table 2.

**Table 2.** **Comparison of emergency treatment time indicators between the two groups of patients (in minutes)**.

Indicator	Waiting time	Diagnosis time	Emergency room waiting time	DNT (Door-to-needle time)*	DPT (Door-to-puncture time)**
Conventional care group (n = 57)	13.43 ± 1.42	12.31 ± 1.74	30.20 ± 2.83	58.33 ± 13.76	92.47 ± 21.83
Comprehensive nursing group (n = 57)	6.32 ± 1.59	7.42 ± 1.85	16.24 ± 2.68	42.17 ± 10.25	71.29 ± 16.94
*t*	25.154	14.523	27.033	7.185	5.963
*p*	<0.001	<0.001	<0.001	<0.001	<0.001

Note: *DNT was analysed only for patients who received intravenous thrombolysis (conventional care: n = 20; comprehensive nursing: n = 22). **DPT was analysed only for patients who received mechanical thrombectomy (conventional care: n = 15; comprehensive nursing: n = 17).

### Post-Pathway Outcomes and Change Scores

#### Post-Pathway Scores at Discharge (T1)

At the T1 assessment, significantly better ESS scores and lower scores on all psychological scales were observed in the comprehensive nursing group compared with the conventional care group (all *p* < 0.001). See Table 3.

**Table 3.** **Comparison of neurological function and psychological state scores at discharge (T1) between the two groups (points)**.

Indicator	ESS pre-discharge score	SAS score	SDS rating	GAD-7 rating	PHQ-9 rating
Conventional care group (n = 57)	72.26 ± 3.99	51.61 ± 5.79	52.37 ± 4.55	8.52 ± 2.83	9.30 ± 3.13
Comprehensive nursing group (n = 57)	89.46 ± 4.41	44.91 ± 5.64	47.79 ± 3.90	5.25 ± 2.11	5.82 ± 2.49
*t*	-21.823	6.257	5.764	6.997	6.556
*p*	<0.001	<0.001	<0.001	<0.001	<0.001

Note: ESS, European Stroke Scale; SAS, Self-Rating Anxiety Scale; SDS, Self-Rating Depression Scale; GAD-7, Generalized Anxiety Disorder-7; PHQ-9, Patient Health Questionnaire-9.

#### Analysis of Change Scores (Δ = T1-T0)

To directly attribute outcomes to the integrated care pathway, change scores were calculated. The improvement in ESS (ΔESS) and the reduction in all psychological scale scores (ΔSAS, ΔSDS, ΔGAD-7 and ΔPHQ-9) were significantly greater in the comprehensive nursing group than in the conventional care group (all *p* < 0.001). See Table 4.

**Table 4.** **Comparison of change scores in neurological and psychological scales**.

Indicator	ΔESS pre-discharge score	ΔSAS score	ΔSDS rating	ΔGAD-7 rating	ΔPHQ-9 rating
Conventional care group (n = 57)	9.92 ± 5.47	-4.57 ± 3.85	-4.70 ± 3.62	-1.56 ± 1.82	-1.93 ± 1.74
Comprehensive nursing group (n = 57)	18.34 ± 6.81	-10.56 ± 4.92	-9.28 ± 4.73	-3.87 ± 2.15	-4.75 ± 2.08
*t*	7.23	7.445	5.892	6.178	7.915
*p*	<0.001	<0.001	<0.001	<0.001	<0.001

Note: ESS, European Stroke Scale; SAS, Self-Rating Anxiety Scale; SDS, Self-Rating Depression Scale; GAD-7, Generalized Anxiety Disorder-7; PHQ-9, Patient Health Questionnaire-9.

#### Adjusted Analyses

To further control for the interference of confounding factors on the research results, in addition to the baseline NIHSS score, the time from onset to admission, hypertension and diabetes, three important confounding factors, namely, age, sex and BMI, were additionally included to fit a multivariate linear regression model. The independent influence of the integrated and optimised nursing pathway on patient outcome indicators was analysed. Multivariable linear regression models, adjusted for baseline NIHSS score, onset-to-door time, hypertension and diabetes, showed that assignment to the comprehensive nursing group remained significantly associated with greater improvement in ΔESS (β [95% confidence interval (CI)] = 8.15 [5.72 to 10.58], *p* < 0.001), greater reduction in ΔGAD-7 (β [95% CI] = -2.18 [-2.92 to -1.44], *p* < 0.001), and greater reduction in ΔPHQ-9 (β [95% CI] = -2.65 [-3.32 to -1.98], *p* < 0.001) (Table 5).

**Table 5.** **Results of the multivariate linear regression analysis of the integrated and optimised the nursing pathways and outcome indicators after adjusting for multiple confounding factors**.

Variable	Adjusted β	95% CI	*p*-value
ΔESS	8.15	5.72 to 10.58	<0.001
ΔGAD-7	-2.18	-2.92 to -1.44	<0.001
ΔPHQ-9	-2.65	-3.32 to -1.98	<0.001

Note: NIHSS, National Institutes of Health Stroke Scale; CI, confidence interval.

### Comparison of Nursing Satisfaction Between the Two Groups

Overall nursing satisfaction was significantly higher in the comprehensive nursing group compared with that in the conventional care group (*p* < 0.001) (Table 6).

**Table 6.** **Comparison of nursing satisfaction (n, %)**.

	Total satisfaction score (mean ± SD)	Not satisfied	Satisfied*
Conventional care group ( n = 57 )	38.42 ± 4.15	17 (29.82)	40 (70.18)
Comprehensive nursing group (n = 57)	45.68 ± 3.27	3 (5.26)	54 (94.74)
χ ^2^			11.885
*p*			<0.001

Note: *Satisfied: overall response of “satisfied” or “very satisfied” on ≥80% of the questionnaire items.

### Independent Predictors of Nursing Satisfaction: Multivariable Logistic Regression Analysis

A multivariable logistic regression model was constructed with the binary satisfaction outcome (Satisfied vs. Not Satisfied) as the dependent variable, adjusting for baseline NIHSS score, onset-to-door time, hypertension and diabetes. Table 7 presents the results. Assignment to the comprehensive nursing group was independently associated with a significantly higher likelihood of achieving a “Satisfied” rating (adjusted odds ratio = 8.915, 95% CI: 2.453 to 32.468, *p* = 0.001). None of the other covariates reached statistical significance as independent predictors in this model.

**Table 7.** **Results of multivariable logistic regression analysis for predictors of nursing satisfaction**.

Variable	Adjusted OR	95% CI	*p*-value
Care pathway (study vs. control)	8.915	2.453 to 32.468	0.001
Baseline NIHSS score	0.953	0.823 to 1.104	0.512
Onset-to-door time (min)	1.002	0.996 to 1.009	0.845
Hypertension (yes vs. no)	1.254	0.413 to 3.814	0.694
Diabetes (yes vs. no)	0.709	0.234 to 2.156	0.546

Note: OR, odds ratio; NIHSS, National Institutes of Health Stroke Scale; CI, confidence interval.

## Discussion

### Summary of Main Findings

The treatment of AIS is highly time-dependent, and starting treatment as early as possible is crucial because the patient’s prognosis will decrease with the increase in treatment time. The short treatment window and the irreversibility of neurological damage determine that the emergency treatment system must pursue maximum efficiency [17, 18]. Studies have found that for AIS patients with large vessel occlusion, saving 1 minute of time from onset to treatment can extend the healthy lifespan by an average of 4.2 days [19]. Traditional sequential emergency workflows—characterised by fragmented coordination, serial testing and passive communication—remain systemic bottlenecks that cannot be resolved by isolated equipment or process adjustments alone. This study demonstrates that an integrated pathway addressing both systemic and psychological bottlenecks is associated with shorter treatment times, better neurological recovery, reduced anxiety and depression and higher satisfaction. These findings extend prior work on process redesign [20, 21] by showing that efficiency gains can be achieved without sacrificing—and indeed while embedding—structured psychological support.

Meanwhile, the acute stress response, serious concerns about the prognosis of the disease, and confusion about complex treatment decisions that are common in AIS patients can induce significant anxiety and depression [22]. Anxiety and depression may lead to decreased adherence to rehabilitation treatment, worsening of functional prognosis, reduced quality of life and increased mortality. Effective management of post-stroke anxiety and depression is necessary to improve the prognosis of patients with AIS [23, 24]. Therefore, constructing a comprehensive care model that integrates technical process optimisation and systematic patient education is of urgent clinical necessity and practical significance to open up the entire chain of AIS treatment and achieve comprehensive care from physiological reperfusion to psychosocial function recovery. Our findings contribute empirical data supporting the feasibility and potential value of such a model. The observed reduction in key time metrics is likely associated with several synergistic redesign elements introduced in the integrated pathway.

### Theoretical Frameworks and Potential Mechanisms

This integrated pathway draws on two complementary frameworks. Social cognitive theory posits that self-efficacy mediates responses to environmental stress. Process optimisation reduced time pressure and uncertainty, lowering cognitive load and enhancing receptivity to education. Structured, phased education—delivered by a nurse coordinator—was designed to strengthen self-efficacy through clear information, modelling and support. The synergy is bidirectional: efficient systems could reduce stress, potentially enabling better learning; educated patients might cooperate more readily, possibly reinforcing efficiency.

The self-regulation model (Leventhal) holds that illness perceptions drive emotional responses. Acute stroke often triggers catastrophic appraisals. Education directly targeted these perceptions by providing accurate information about stroke, treatment and recovery, reducing uncertainty and perceived threat. Process optimisation indirectly shortened the unknown phase and signalled competence.

Although component effects cannot be disentangled, the observed associations align with this theoretically grounded, synergistic model.

### Positioning within the Literature and Implications for Patient-Centred Care

Regarding the pursuit of treatment efficiency, the magnitude of time reduction achieved in our study aligns with the effects reported in recent initiatives focused on systematic process re-engineering, such as regional stroke network optimisations [20] and enhanced emergency medical services protocols [21]. This consistency validates the core principle that parallel processing and team-based activation are effective strategies for conquering time-based barriers. However, these prior studies predominantly targeted “hard” time metrics as primary endpoints. Conversely, in the domain of post-stroke psychological support, robust evidence exists—as exemplified by the video-based education intervention for stroke survivors by Tjokrowijoto *et al*. [11]—demonstrating that structured psychoeducation can effectively alleviate emotional distress. A common feature of such interventions, however, is their delivery during the post-stabilisation or rehabilitation phase, potentially missing the acute stress window. The distinct advance of the present study lies in its systematic integration and concurrent evaluation of these two critical dimensions—minute-by-minute biological clock management and patient-experience-centred psychosocial support—within the hyperacute emergency setting itself. This approach suggests that an optimised stroke pathway may not only salvage the ischaemic penumbra but also mitigate the psychological distress inherent to the chaotic and uncertain emergency experience. By addressing the “clock” and the “person” from the first moment of care, our model offers a practical iteration of the patient-centred comprehensive care paradigm for acute stroke. This integrated model aligns with the emerging concept of psychologically informed medical environments, which emphasise addressing systemic efficiency and patient emotional experience in acute care settings.

The observed improvements in efficiency and psychological outcomes can be interpreted through complementary theoretical lenses. Firstly, from a systems perspective, the observed reduction in key time metrics likely stems from several synergistic redesign elements introduced in the integrated pathway. Traditional emergency room models often suffer from delays and information gaps in pre-hospital and in-hospital coordination, multi-departmental collaboration and patient communication. The optimisation scheme promoted in this study primarily aims to break down the barriers of traditional emergency room procedures and construct a time-centric, parallel and standardised treatment network. By integrating pre-hospital early warning and in-hospital one-click activation mechanisms, real-time transmission of patient information and rapid response from the treatment team are achieved. The implementation of a parallel process that simultaneously conducts examinations, blood sampling and clinical assessments significantly reduces unnecessary waiting times. Moreover, establishing a green channel for “treatment first, payment later” effectively avoids treatment delays caused by cost issues. These systemic improvements collectively constitute the direct technical reason for the significant reduction in waiting time and diagnosis time achieved by the comprehensive nursing group.

Shorter recanalisation times are known to salvage the ischaemic penumbra and limit infarct expansion [25]. The use of ESS—sensitive to functional recovery—allowed detection of this biological benefit.

The observed reduction in anxiety and depression is likely associated with synergy between efficiency-driven stress reduction and structured psychoeducation. Phased education delivered by a dedicated nurse coordinator—emergency, inpatient and pre-discharge—reduces uncertainty, enhances perceived control and builds trust [7, 26]. Unlike prior interventions delivered post-stabilisation [5, 11], this model embeds support within the hyperacute phase, targeting distress at its peak. These benefits may be interdependent: efficient care could reduce cognitive load, potentially enabling better information processing; informed patients might cooperate more readily, possibly reinforcing system flow.

Importantly, the effects of process optimisation and health education within this integrated model are intertwined, and this study cannot disentangle their individual contributions. The observed benefits are likely synergistic: streamlined care reduces physiological stress and time pressure, while structured education concurrently addresses psychological distress. Our findings advocate for an integrated emergency care model that concurrently addresses system efficiency and patient experience. Traditional approaches often target these aspects in isolation—focusing either on time metrics or on later psychological support. By redesigning the care pathway to simultaneously reduce delays and address the fear and confusion commonly experienced by patients, our model aligns with the principles of patient-centred comprehensive care [4, 9]. This finding suggests that embedding psychosocial support into the initial design of acute stroke protocols is feasible and may enhance both clinical and subjective outcomes. Future research should employ prospective designs to establish causality and explore the model’s mechanisms and long-term impact through mixed-methods approaches.

Post-stroke psychological distress has gained increasing research attention, with particular focus on its mechanisms, risk factors and the development of psychosocial nursing interventions to improve quality of life [27, 28]. Patients with AIS face especially high psychological stress because of the sudden onset and disabling nature of the condition, resulting in significantly elevated rates of anxiety and depression compared to the general hospitalised population [29]. Routine unstructured verbal communication often fails to adequately address the helplessness and fear stemming from information deficits. By contrast, the phased, structured health education embedded in our integrated pathway—tailored to patients’ evolving needs across emergency, inpatient and pre-discharge phases—provided systematic, progressive disease knowledge and treatment guidance. This approach reduced doctor-patient information asymmetry, enhanced patients’ perceived control and fostered positive rehabilitation expectations by improving their understanding of the condition and treatment trajectory. Improving illness perception and sense of control is a well-established mechanism for reducing anxiety in medical contexts. The significant reductions in GAD-7 and PHQ-9 scores observed in this study align with prior evidence on psychoeducation in stroke and support the psychological value of structured information delivery within a comprehensive care model. Of note, the effects of process optimisation and health education in this integrated model are interdependent and cannot be disentangled. The observed benefits are likely synergistic, with streamlined care reducing physiological stress and time pressure while structured education concurrently addresses psychological distress.

However, this study has certain limitations. As a retrospective cohort study, it is susceptible to potential biases such as time trends and unmeasured confounding variables. Even after balancing baseline characteristics and adjusting for multiple variables through regression analysis, residual biases cannot be completely eliminated, and causal inferences cannot be established. The patient selection process was not detailed, and the nursing satisfaction questionnaire was not formally tested for construct validity in a larger AIS population. The extrapolation and strength of the argument of the results are thus limited.

## Conclusions

This retrospective study shows that the comprehensive nursing model combining emergency process optimisation and structured anxiety and depression health education can significantly shorten the diagnosis and treatment time of patients with AIS, improve neurological function, alleviate anxiety and depression and enhance nursing satisfaction. It provides preliminary evidence for the clinical construction of a patient-centred comprehensive emergency model.

## Availability of Data and Materials

The datasets used and/or analysed during the current study were available from the corresponding author on reasonable request.
